# The T/Tn-Specific *Helix pomatia* Lectin Induces Cell Death in Lymphoma Cells Negative for T/Tn Antigens

**DOI:** 10.3390/cancers13174356

**Published:** 2021-08-28

**Authors:** Mathias Simplicien, Annick Barre, Yamina Benkerrou, Els J. M. Van Damme, Pierre Rougé, Hervé Benoist

**Affiliations:** 1 UMR 152 PharmaDev, Institut de Recherche et Développement, Faculté de Pharmacie, Université Paul, Sabatier, F-31062 Toulouse, France; mathias.simplicien@univ-tlse3.fr (M.S.); annick.barre@univ-tlse3.fr (A.B.); yamina.benkerrou@gmail.com (Y.B.); pierre.rouge.perso@gmail.com (P.R.); 2 Department of Biotechnology, Faculty of Bioscience Engineering, Ghent University, B-9000 Ghent, Belgium; elsjm.vandamme@ugent.be

**Keywords:** lectins, T/Tn antigens, T lymphoma, truncated *O*-glycans, cancer cell death, *Helix pomatia* agglutinin, Morniga G, leukemia, biomedical applications

## Abstract

**Simple Summary:**

Changes in glycosylation, such as incomplete synthesis and higher density of *O*-glycans on the cell surface, are frequently observed in cancer cells. Several types of truncated *O*-glycan structures, e.g., T/Tn antigens, are suspected to disrupt molecular interactions between tumor microenvironment and immune cells, for instance, facilitating cancer immune-escape. Therefore, numerous exogenous lectins targeting aberrant *O*-glycans are interesting tools for cancer diagnosis, prognosis, and therapy. However, the ability of exolectins to detect subtle alterations in the glycome of tumor cells and to interfere in tumor/healthy cell interactions remains largely unknown. The present article reports for the first time that the *Helix pomatia* (HPA) lectin, a well-known T/Tn-specific lectin, currently used as a tool in cancer diagnostics, kills Tn-positive leukemia cells and Tn-negative lymphoma cells but does not affect healthy lymphocytes. Thus, HPA could be used to discriminate between tumor and healthy cells, and detect subtle alterations in the glycosylation profile.

**Abstract:**

Morniga G is a T/Tn-specific lectin, inducing cell death in Tn-positive leukemias but not in healthy lymphocytes. *Helix pomatia* lectin (HPA) is another T/Tn-specific lectin, currently used as tool for cancer diagnostics. The HPA-mediated tumor cell death was evaluated on human leukemia and mouse lymphoma cells, and compared to the effect of Morniga G. Both lectins induced an equivalent percentage of cell death in Tn-positive Jurkat human leukemia. In contrast, EL4 mouse lymphoma resisted Morniga G-mediated cytotoxicity but were killed by HPA at concentrations of 2.5 μg/mL (0.032 nM) and higher. In both malignant cells, HPA-mediated cell death showed features compatible with apoptosis (annexin-externalization, caspase-activation, mitochondrial membrane depolarization, and ROS production). Cytometry analysis indicated that EL4 cells are T/Tn-negative. Because previous results showed a high amount of *N*-acetylgalactosamine (GalNAc, sugar present in Tn antigen) on EL4 cell surface, this GalNAc could be involved in the formation of truncated *O*-glycans other than the T/Tn residues. When compared to Morniga G, bioinformatic analysis suggested that HPA benefits from an extended carbohydrate-binding site, better adapted than Morniga G to the accommodation of more complex branched and truncated *O*-glycans (such as core 2). Finally, HPA killed EL4 cells but not healthy lymphocytes in a mixture of lymphoma cells + lymphocytes, suggesting that HPA selectively triggers tumor cell death.

## 1. Introduction

Dramatic changes in the glycosylation profile of membrane molecules are one of the obvious characteristics of cancer cells [1,2]. These changes involve glycolipids and glycoproteins, for instance the *N*- and/or *O*-glycosylation of proteins. Among the possible modifications, three main changes are frequently observed in proteins: (i) an increase of *N*-glycan branching mediated mostly by β1,6-*N*-acetylglucosamine transferase 5 (MGAT5) [3], (ii) an aberrant *O*-glycan synthesis resulting in the expression of a large quantity of truncated glycans on the tumor cell membranes, such as the over-expression of Tn antigen (GalNacαSer/thr) [4,5,6], and (iii) a hypersialylation [7]. The presence of aberrant glycosylation can be demonstrated in a majority of cancers, both in solid tumors and in hematological malignancies, e.g., [8,9,10]. Some changes in the glycan patterns are used as diagnostic or prognostic markers [7,8,9,11] and some can serve as therapeutic targets [1,7,12].

The presence of aberrant glycosylation is one of the consequences of the genetic and epigenetic abnormalities that accompany carcinogenesis [2]. The alteration of the cancer cell glycosylation is not only a result of the pathological cancer processes, it can have functional consequences strongly implicated in cancer pathophysiology. Some of these alterations participate in tumor growth and migration, the appearance of metastasis, and the escape of tumors to anti-cancer immune response [5,7,13]. Thus, certain modifications of glycosylation could facilitate the cancer-cell resistance to death induced by various immunological effectors (e.g., cytokines and killer cells). For instance, alterations of the *N*-glycosylation of Fas ligand receptor (CD95) or the *O*-glycosylation of Trail-receptor (TRAIL-R1 and TRAIL-R2) could modulate the tumor cell death triggered by FasL or TRAIL, respectively, thus participating in tumor escape. For instance, the normal *O*-glycosylation of TRAIL-R2 (DR5) [14,15,16] and the *N*-glycosylation of TRAI-R1 (DR4) [17] seem essential for efficient triggering of TRAIL-mediated apoptosis. Consequently, alterations in the glycosylation of TRAIL/FasL receptors present on the tumor cell surface could be involved in the dysregulation of immune surveillance against tumor cells.

In healthy conditions, animal glycome participates in tissue homeostasis. Many molecules are able to interact with glycome and help to maintain homeostasis. Emerging evidence indicates that glycan molecules encode biological information that can be recognized and translated with use of glycan-binding proteins (GBPs). Lectins are a major group of GBPs characterized and defined by the presence of carbohydrate-recognizing domains (CRDs). To date, in animals and humans, several endogenous lectins have been clearly implicated in a variety of physiological and pathological processes, e.g., the C-type lectins, the galectins, and the I-type lectins (Siglecs and others) [18,19]. 

Among GBPs, the plant lectins are exogenous molecules that can specifically recognize glycan patterns in animals, and have been considered for a long time as useful glycan probes to analyze healthy or pathological glycan patterns, e.g., for the diagnosis or prognosis of cancer [20,21]. Thus, several plant lectins specifically recognize aberrations in the glycosylation profile expressed by cancer cells and could be used to analyze the functional role of glycosylation in cancer cells [22,23,24]. In addition, because of their capacity to recognize specific glycans, plant lectins have been proposed as tools for targeting drugs towards pathological glycan patterns [12,25]. Numerous observations indicate that some plant lectins can induce cell death in cancer cells, only because of their binding to glycans on cell membrane glycoproteins or glycolipids, which in turn can trigger cell death pathways [15,20,24,26], and not because of their intrinsic toxicity (in contrast to ricin and some other lectins from the R-type family, where the lectin subunits are known to be associated to a separate subunit possessing a potent intrinsic toxin activity). However, although lectin binding on the cells is necessary to induce cell death, it is not sufficient to provoke apoptosis, suggesting that the lectin effects on cells depend, at least partly, on the cell membrane protein (or the lipid) carrying the glycans recognized by the lectin. 

*O*-glycans are present on many glycoproteins. This posttranslational modification is characterized by the attachment of one GalNAc to a Ser or Thr, followed by the formation of linear or branched chains with variable length and composed of different sugars, except for Man and Glc. T (Galβ1-3GalNAcαSer/Thr) and Tn (GalNAcαSer/Thr) glycotopes are among the most abundant truncated *O*-glycans observed on tumor cells. The T/Tn glycotopes, together with other *O*-glycans aberrantly overexpressed on cancer cells, represent cancer-markers as well as potential therapeutic targets [4,5,6,12].

Among the numerous plant lectins known to recognize Tn antigen, Morniga G is a tetrameric lectin belonging to the subfamily of galactose-binding jacalin-related lectins isolated from black mulberry (*Morus nigra*) [27].

H-type lectins are a group of exogenous lectins occurring in invertebrates, such as *Helix pomatia*, but also present in higher fungi and microorganisms. These lectins are mainly GalNAc-specific. The *Helix pomatia* agglutinin (HPA) is a hexameric glycoprotein known to be Tn-specific. HPA binding to cancer cells can be associated with metastatic invasion and poor patient prognosis [28].

Recently we demonstrated that Morniga G can interact with Tn antigen present on Jurkat human leukemia, inducing tumor cell death but not death of Tn-negative healthy peripheral blood lymphocytes, at least partly via the TRAIL/DR5-dependent pathway [15]. The increase of Tn epitopes on strategic molecules such as DR5 could be involved in the resistance of tumor cells to TRAIL-induced cell death [14,15,16,17].

The lectin from *Helix pomatia* is used in diagnostics for several human carcinomas [11,29,30,31,32]. However, to our knowledge very little information is known about the putative functional role of HPA-binding glycoproteins on tumor cells. In the present paper, we demonstrate for the first time that HPA induces cell death in human Jurkat T-cell leukemia and mouse EL4 T-cell lymphoma. In the mouse, HPA is clearly more toxic than Morniga G for EL4 cells. In addition, HPA appears to be non-toxic for healthy lymphocytes.

## 2. Results

### 2.1. HPA Kills In Vitro the Tn-Positive Jurkat T-Cell Leukemia

**Figure 1:**
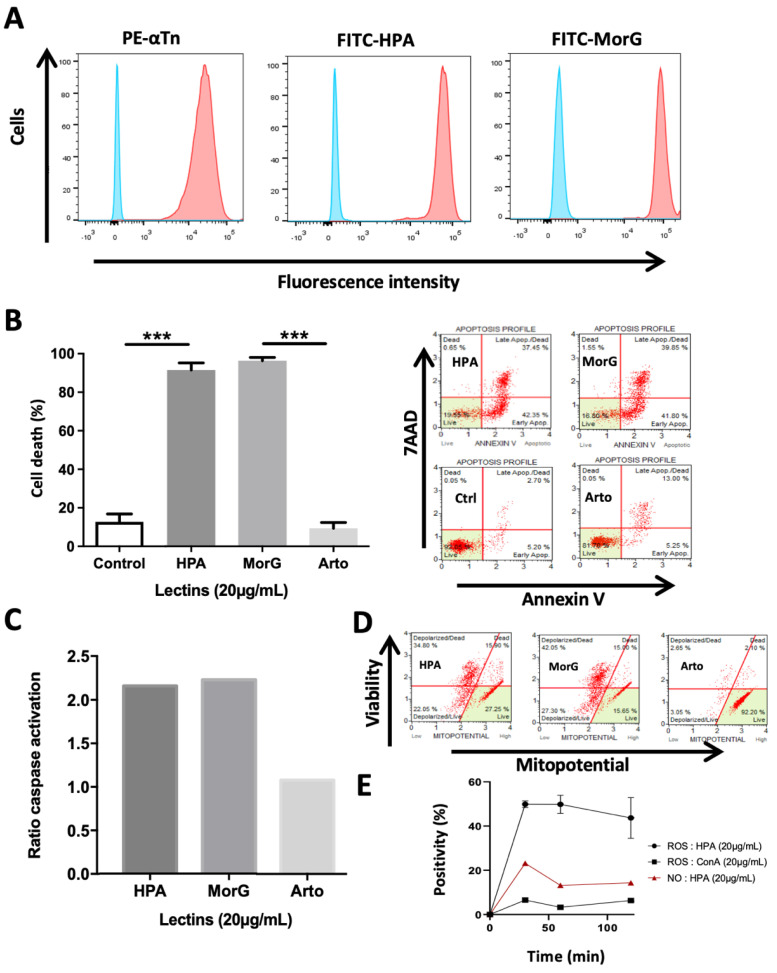
HPA induces cell death in human T-leukemia cells: (**A**) Jurkat A3 leukemia cells were labeled with anti-Tn mouse monoclonal antibody (PE-αTn), FITC-conjugated HPA (FITC-HPA), or FITC-conjugated Morniga G (FITC-MorG), and analyzed using cytofluorimetry (blue curves indicate cell autofluorescence). (**B**–**D**) Leukemia cells were cultured for 48 h in the presence of 20 μg/mL HPA, MorG, or Artocarpin (Arto), then analyzed by cytofluorimetry. (**B**) Specific cell death evaluated using Annexin V/7AAD assay, on the right: representative experiment; (**C**) caspase activation; and (**D**) mitochondrial depolarization, representative of two independent experiments. (**E**) Jurkat cells were cultured with 20 μg/mL HPA or Concanavalin A (ConA). Next, reactive oxygen species (ROS) or nitric oxide (NO) were evaluated at different times using cyto-fluorimetry. (**B**,**C**,**E**), Results are means ± SD of at least three independent experiments or means of two independent experiments. Significant differences are indicated (*** *p* < 0.001).

To explore the possibility that HPA activates cell death pathways, its toxic effect on Jurkat Tn-positive cells was evaluated and compared to the effect of MorG, previously demonstrated to be able to kill Jurkat cells. As expected, HPA binds to Jurkat T-cells with similar efficiency to Morniga G, as observed in cytometry analysis (Figure 1A). More interesting, using previous experimental conditions defined to induce optimal Morniga G-mediated cell death in Jurkat leukemia, HPA triggers similar cell apoptosis (Figure 1B), caspase activation (Figure 1C), and depolarization of mitochondrial membrane potential (Figure 1D). However, the lectin-mediated toxicity on T leukemia cells was not observed with the mannose (Man)-specific lectin Artocarpin (Figure 1B–D), in spite of its binding to Jurkat T-cells. Finally the ROS production was evaluated, and compared to that observed during T-cell activation by ConA. ConA is a Glc/Man-specific lectin known to cross-link T-cell receptors and activate T cells, such as Jurkat cells. HPA already triggers a strong ROS production after 30 min incubation with tumor cells, and this effect is higher than the ROS-induction mediated by ConA (Figure 1E).

### 2.2. HPA Kills T/Tn-Negative EL4 T-Lymphoma Cells

**Figure 2:**
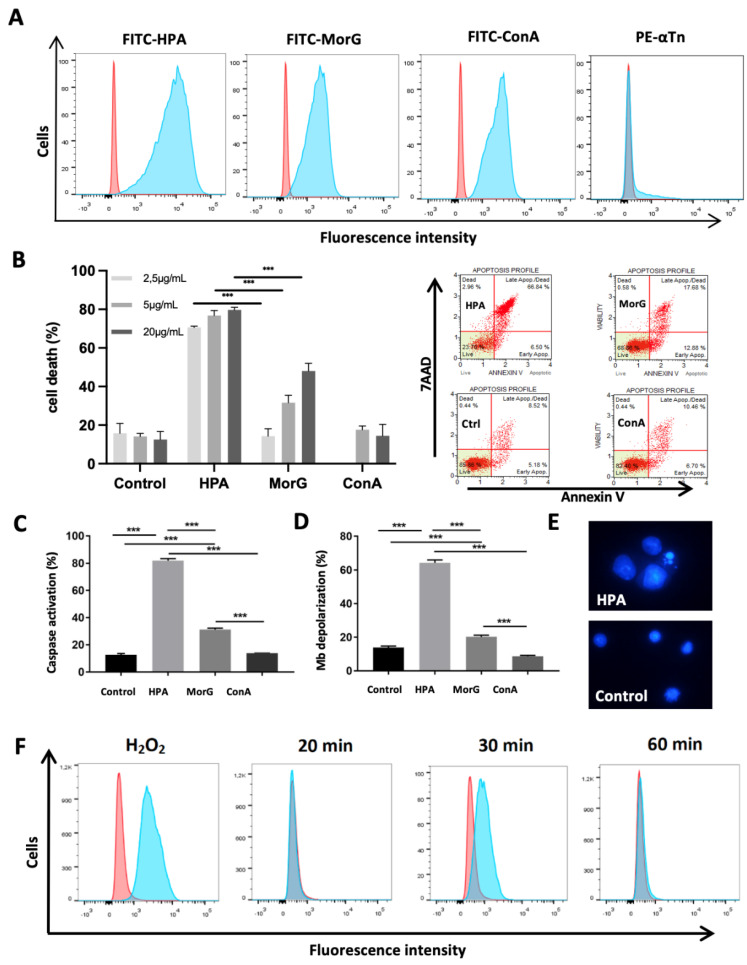
HPA induces cell death in mouse T-lymphoma cells: (**A**) EL4 lymphoma cells were incubated with anti-Tn mouse monoclonal antibody + PE anti-mouse antibody (PE-αTn), FITC-conjugated HPA (FITC-HPA), FITC-conjugated Morniga G (FITC-MorG), or FITC-conjugated ConA (FITC-ConA) and analyzed using cytofluorimetry (Red curves indicate autofluorescence). (**B**–**D**) EL4 lymphoma cells were cultured for 48 h with different concentrations of HPA, MorG, and ConA and analyzed using cytofluorimetry: (**B**) cell viability evaluated with Annexin V/7AAD assay (on the right, representative experiment), (**C**) caspase activation, and (**D**) mitochondrial membrane depolarization (**C**,**D**, lectin 5 μg/mL). (**E**) Cellular morphology was observed after DAPI staining and using fluorescent microscopy, representative of three independent experiments. (**F**) EL4 cells were incubated for 20–50 min with 20 μg/mL HPA. Next, reactive oxygen species production was evaluated at different times using dihydroethidium assay in cytofluorimetry, as compared with H_2_O_2_ treatment (0.1 mM, 20 min). (**B**–**D**), results are means ± SD of at least three independent experiments. Significant differences are indicated (*** *p* < 0.001).

Recently it was demonstrated that murine EL4 cells exhibited higher levels of GalNAc than normal T cells [33], suggesting that these cells could be possible targets for HPA and Morniga G. Cytometry analysis indicated that both lectins bind to EL4, similarly to the Glc/Man-specific lectin ConA (Figure 2A). However, no staining of EL4 was observed with anti-Tn (anti-GalNAcαSer/Thr antigen) monoclonal antibody (Figure 2A). Analysis of the concentration-dependent effect in in vitro experiments indicated that only HPA induced significant EL4 cell death at a concentration of 2.5 μg/mL after 48 h incubation. Data were compared to Morniga G, used as a T/Tn-specific lectin control, and ConA, used as a negative control (Figure 2B). In addition, HPA-treated cells showed classical apoptosis features such as annexin positivity (Figure 2B), caspase activation (Figure 2C), mitochondrial-membrane potential depolarization (Figure 2D), and characteristic morphological alterations (e.g., nuclear fragmentation and condensation) (Figure 2E). In addition, an increase in cell size was observed in some HPA-treated cells, suggesting that the lectin binding could also induce different cell-death pathways simultaneously in the EL-4 cell population (Figure 2E). Finally HPA treatment induced a high ROS signal in EL4 cells, which was compatible with triggering of various processes of programmed cell death (Figure 2F). In conclusion, all the results indicate that the Gal/GalNAc-specific HPA provokes strong cell death in EL4 cells as compared to Morniga G, another Gal/GalNac-specific lectin, whereas ConA does not kill cells in spite of strong binding to the tumor cells.

### 2.3. HPA Induces Death in EL4 T-Cells but Not in Healthy T-Cells

**Figure 3:**
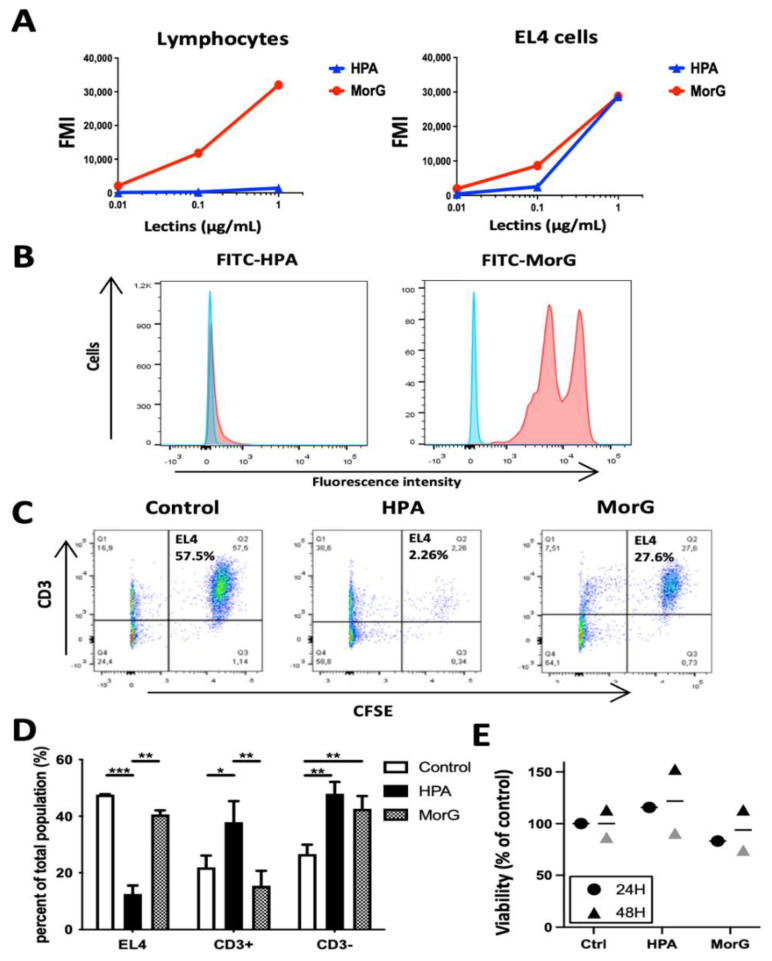
HPA induces a discriminative toxicity on EL4 tumor cells: (**A**,**B**) Non-adherent splenocytes from healthy mice (lymphocytes) or EL4 lymphoma cells were labeled by increasing concentrations of FITC-conjugated HPA (FITC-HPA) or FITC-conjugated Morniga G (FITC-MorG) and analyzed using cytofluorimetry. (**B**) Representative staining of healthy lymphocytes using 1μg/mL FITC-HPA or FITC- MorG. (**C**,**D**) Mixture (1:1) of healthy lymphocytes and CFSE-labeled EL4 lymphoma cells were cultured for 48 h in the presence of 20 μg/mL HPA or MorG, washed and then incubated with PE-labelled anti-CD3 mouse monoclonal antibody and analyzed using cytofluorimetry: (**C**) representative experiment and (**D**) means of three independent experiments. (**E**) Lymphocytes from healthy mice were incubated for 24–48 h with 20 μg/mL HPA or MorG, then cell viability was evaluated using cytofluorimetry. Rounds and triangles are means of three to four mice. Horizontal lines are means of the two independent experiments after 48 h cultures. Significant differences are indicated (* *p* < 0.05; ** *p* < 0.01; *** *p* < 0.001).

Because Morniga G was shown to bind strongly to the Tn-positive Jurkat T-cell leukemia but not to healthy human peripheral blood lymphocytes [15], the binding of 0.01 to 1 μg/mL HPA was evaluated on murine lymphocytes (non-adherent splenocytes, i.e., NK, B, and T lymphocytes) and compared to EL4 cells. HPA did not stain NK, B, or T lymphocytes (or very faintly at 1 μg/mL concentration, Figure 3A), whereas Morniga G actually stained two distinct lymphocyte populations (Figure 3B). When a mixture of non-adherent splenocytes + EL4 cells was treated with HPA or Morniga G (20 μg/mL, 48 h), followed by cell death analysis using cytometry, it was observed that HPA killed EL4 cells more efficiently than Morniga G, whereas percentages of CD3+ and CD3- lymphocytes were increased significantly as compared to the control, likely mainly due to EL4 cell death. In the Morniga G-treated-mixture cells, only the percentage of CD3-negative lymphocytes increased, suggesting that HPA and Morniga G have discrepancy effects on healthy lymphocytes (Figure 3C,D). Finally, HPA did not alter the viability of healthy lymphocytes after 24–48 h culture (Figure 3E). Altogether, the results suggest that HPA efficiently discriminates between EL4 lymphoma cells and healthy lymphocytes, indicating that the lectin can recognize glycan structures on tumor cells which are under-expressed or absent on normal mouse lymphocytes.

### 2.4. What Could Be the Targets of HPA on EL4 T-Lymphoma Cells?

**Figure 4:**
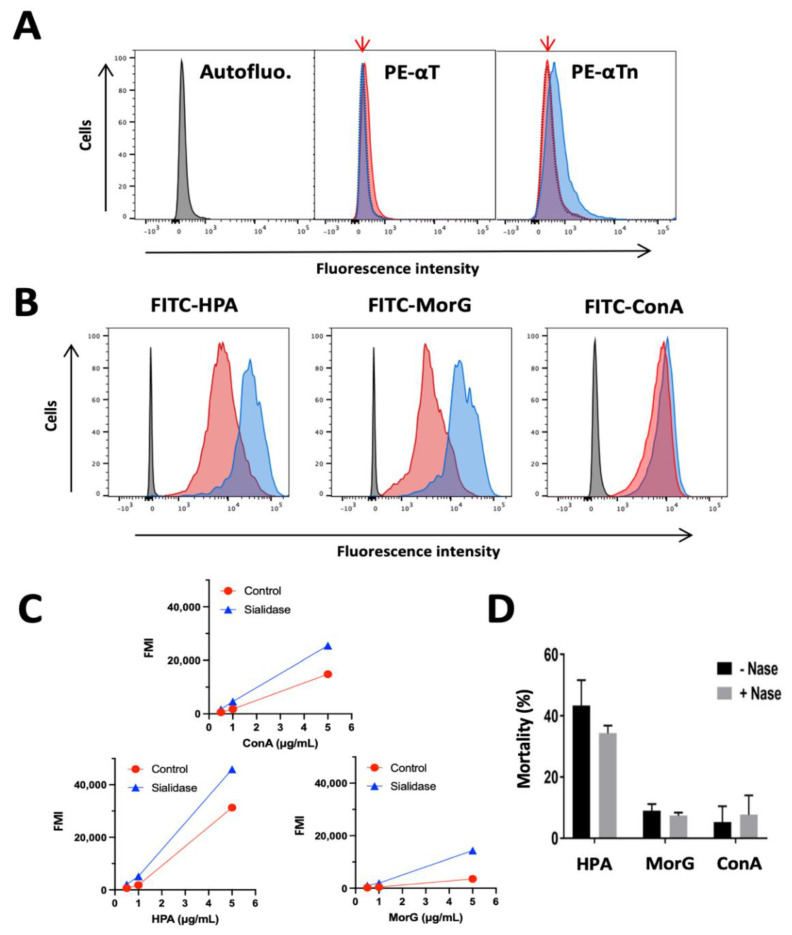
Effect of neuraminidase treatment on glycan detection at EL4 cell surface and lectin-induced cell death: (**A**–**C**) EL4 cells were incubated with (blue) or without (red) neuraminidase (20 mUI, 30 min, 37 °C) and labeled: (**A**) with anti-Tn (PE-αTn) or anti-T (PE-αT) mouse monoclonal antibodies or (**B**,**C**) with FITC-lectins: HPA (FITC-HPA), MorG (FITC-MorG), or ConA (FITC-ConA). (**A**) Red arrow indicates autofluorescence pic position (grey curve); (**B**) representative results of two independent experiments with 5 μg/mL of labeled lectin, with blue curve corresponding to neuraminidase-treated cells; and (**C**) lectin concentration effect on labelling. (**D**) EL4 cells were incubated with (+ Nase) or without (− Nase) neuraminidase, washed, and cultured for 48 h with 2.5 μg/mL of HPA, MorG, or ConA, then cell death was analyzed using cytofluorimetry. Results are means ± SD of three independent experiments.

It was previously demonstrated that sialic acids are strongly expressed on EL4 cells as compared to normal T-cells [33]. To evaluate the putative role of sialic acids in HPA-induced cell death, the effect of sialidase treatment of EL4 cells was first evaluated with respect to binding of anti-T/Tn Mabs, HPA, Morniga G and ConA to EL4 cells (Figure 4A–C). Firstly, using cytometry analysis no staining of EL4 was observed with anti-T (anti-core 1 or anti-Galβ1-3GalNAcαSer/Thr) antibody, indicating that EL4 cells are T negative cells (Figure 4A) and that core 1 is not a target for HPA. Secondly, the anti-Tn Mab binding was moderately increased by neuraminidase-treatment, suggesting that EL4 cells express sialyl-Tn or that sialic acids hinder the access of anti-Tn Mabs to Tn antigen (Figure 4A). In addition, as expected the binding of the three lectins was increased after sialidase treatment (Figure 4B,C). Finally the effect of sialidase pre-treatment was evaluated on lectin-induced cell death, but revealed no increase in cell death (Figure 4D), suggesting that sialic acid does not significantly alter the HPA-induced toxicity against EL4 T lymphoma.

**Figure 5:**
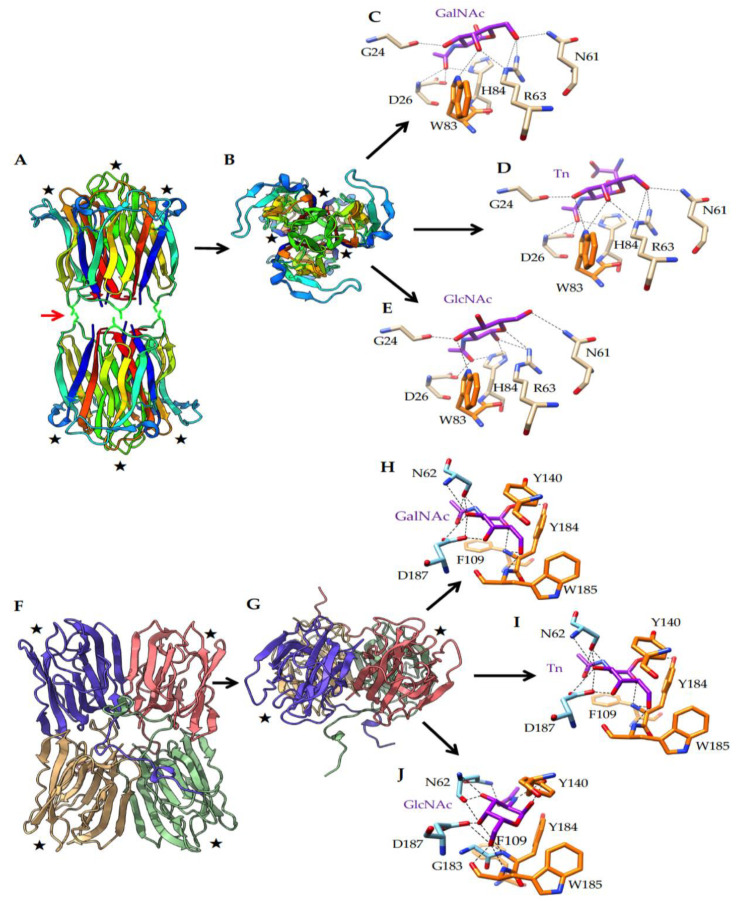
Comparative bioinformatic modeling of HPA and Morniga G: (**A**) Ribbon diagram of the overall three-dimensional structure of HPA hexamer, built from two homotrimers associated by three disulfide bridges. The cysteine residues involved in the association of the homotrimers in a hexameric structure are indicated by the red arrow. Black stars indicate the localization of the CBSs at the top of each homotrimer. (**B**) Front face of the HPA homotrimer showing the localization of the CRDs. (**C**–**E**) Docking of GalNAc (**C**), Tn antigen (**D**), and GlcNAc (**E**) to the monosaccharide-binding site (MBS) of HPA through a network of hydrogen bonds (back dashed lines) connecting hydrophilic amino acid residues forming the MBS to the sugars GalNAc and GlcNAc, and the sugar derivative Tn antigen. Aromatic residues (colored orange), located in the vicinity of the MBS, reinforce the interaction with the sugars by stacking interactions with the pyranose ring of the sugars. (**F**) Ribbon diagram of the overall three-dimensional model Morniga-G tetramer, built from the non-covalent association of four monomers, each containing one MBS (black star) located at the top of beta-prism monomer. (**G**) Front face of the Morniga-G homotetramer showing the localization of the CRDs. (**H**–**J**) Docking of GalNAc (**H**), Tn antigen (**I**), and GlcNAc (**J**) to the MBS of Morniga-G, via a network of hydrogen bonds (back dashed lines) connecting hydrophilic amino acid residues forming the MBS to the sugars GalNAc and GlcNAc, and the sugar derivative Tn antigen. Aromatic residues (colored orange), located in the vicinity of the MBS, reinforce the interaction with the sugars through stacking interactions with the pyranose ring of the sugars.

**Figure 6:**
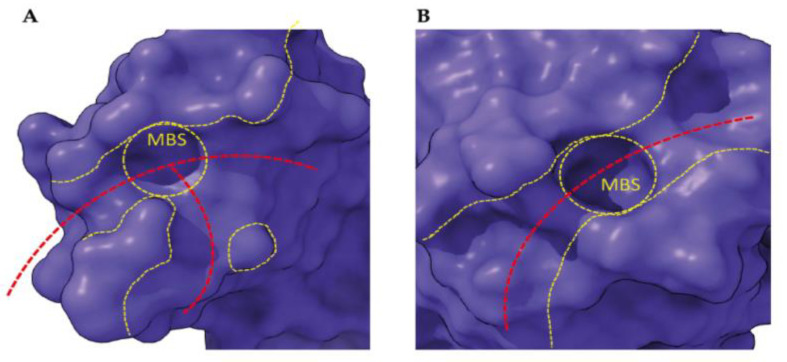
Molecular surface of the top of HPA (**A**) and Morniga G (**B**) monomer: The topographical organization of the surface surrounding the monosaccharide-binding site/pocket (delineated by a yellow dashed circle) is shown. The red dashed lines define the axes along which additional sugar units linked to the sugar occupying the monosaccharide-binding site (MBS) could be accommodated by hydrophilic and hydrophobic residues forming the extended CBS located around the MBS. The regions surrounding the MBS susceptible to accommodating branched *O*-glycans are delineated by yellow dashed lines.

Since previous data indicated that HPA can be GlcNAc-specific [34], a comparative bioinformatic analysis was performed to evaluate the GlcNAc binding capacity of HPA and Morniga G. GlcNAc appeared as a possible ligand for both lectins and results were very similar to GalNAc or Tn antigen (Figure 5). Because all above results indicated that Morniga G and HPA show different effects on lymphocytes and lymphoma cells (e.g., in their capacity to bind to lymphocytes and to induce cell death in EL4 cells), the structural differences between the two lectins are worth investigating, in order to find an explanation for the discrepancies observed. Although both lectins exhibited a very similar binding towards GalNAc and Tn antigen, they differed in their overall organization, the number and localization of the CRDs, and the topography of the molecular surfaces surrounding their monosaccharide-binding sites (MBS) (Figure 5). HPA consists of two homotrimers, each possessing three CRDs resulting in six CRDs (hexavalent lectin, [35]) (Figure 5A), whereas Morniga-G is a homotetramer that only contains four CRDs (tetravalent lectin) (Figure 5F). HPA contains three CRDs in each homotrimer, located close to each other (≈27 Å) at both ends of the lectin. In contrast CRDs are more separated in Morniga-G (≈45 Å) (Figure 5B,G). Accordingly, HPA should be better adapted than Morniga-G to induce the formation of TACA clusters on the cell surface. In addition to the recognition of simple sugars, the recognition of more complex *O*-glycans (or *N*-glycans) by HPA benefits from an extended carbohydrate-binding site, better adapted to the accommodation of branched *O*-glycans, compared to Morniga-G (Figure 6). In fact, the topography of the areas distributed around the MBS in HPA, offers more possibilities for a better accommodation of, for instance, α1,2-, α1-4, or α1-6-branched *O*-glycans due to the largely open configuration of the molecular surfaces surrounding the MBS (Figure 6A). In contrast, the molecular surfaces around the MBS of Morniga G are oriented on a single axis that prevents heavily branched *O*-glycans being properly accommodated by the extended binding site of the lectin [36] (Figure 6B). All these discrepancies occurring between both HPA and Morniga G, should account for a rather distinct behavior and binding to glycan structures on cancer cells.

## 3. Discussion

In this paper we demonstrated for the first time that the *Helix pomatia* lectin, commonly used in the diagnosis of various cancers [11,29,30,31,32], can kill human T lymphocyte leukemia and mouse T lymphoma. Using the human Jurkat cell leukemia, the level of HPA-induced cell death was similar to that with Morniga G-induced cell death. Morniga G was previously demonstrated to induce cell death in cell leukemias expressing Tn antigen but not in healthy lymphocytes [15], probably because naive or resting T-lymphocytes are Tn negative [37,38]. In the Jurkat human T cell leukemia, HPA could trigger cell death pathways similar to Morniga G, most likely after interaction with membrane glycoproteins carrying altered *O*-glycans, i.e., Tn antigen, strongly expressed by Jurkat T cells [14,15] but not by healthy resting T lymphocytes [15,38]. Interestingly, 2.5 μg/mL (0.032 nM) HPA is clearly more efficient than same concentration of Morniga G to induce cell death in mouse T lymphoma (Figure 2B and Figure 4D), and this result was confirmed in assays with a mixture of EL4 T lymphoma cells + normal lymphocytes (Figure 3C,D). Furthermore, HPA and Morniga G induce different quantitative modifications in CD3-positive and -negative lymphocyte populations (Figure 3D), indicating that the two lectins displaying similar sugar-binding specificities towards simple sugars (i.e., to the GalNAc) might present various biological effects on tumor cells and normal cells, at least partly because the two lectins could exhibit distinct binding affinities with different oligosaccharides incorporating GalNAc (or galactose). However, a role for structural differences between HPA and Morniga G cannot be excluded. Indeed, HPA is a hexameric lectin with six possible CRDs [35]), whereas Morniga G is a tetrameric lectin with only four CRDs [36]. Evidently, a lectin molecule with a higher number of CRDs can interact with more membrane glycoproteins and this could be an advantage for cell signal induction by facilitating receptor clustering, lattice formation on the cell surface, and triggering of downstream signaling, for instance to induce cell death.

As indicated above, both HPA and Morniga G are known as Tn-specific lectins. Consequently, HPA-induced cell death in Jurkat leukemia could be due to lectin binding to Tn antigen overexpressed on the cell surface, and at least partly due to the Tn-antigen-modified DR5 (TRAIL receptor) as previously shown for Morniga G [15]. In agreement with the programmed cell death characteristics observed in the present study (Figure 1B–D), it can be speculated that the induction of apoptosis by HPA is possibly due to DR5 clustering triggered by lectin binding to the surface glycans of Jurkat cells.

It was previously demonstrated that EL4 cells exhibit higher levels of sialic acids on the cell membrane compared to normal T cells [33]. Our results indicated that HPA-induced cell death is not significantly affected by the presence of sialic acid on EL4 cells, in spite of the fact that moderate inhibition of HPA-binding to the EL4 cells correlated with the surface hypersialylation, suggesting that HPA-mediated cell death is largely independent of the presence of sialic acid.

In addition, the present results indicated that the aberrant synthesis of large quantities of T/Tn antigens by EL-4 lymphoma must be ruled out, suggesting that an increase of *O*-glycan Core 1 (i.e., T antigen), cannot be the target for HPA on the EL4 cell membrane. However, other truncated *O*-glycan alterations could be possible targets for HPA, resulting in induction of cell death. For instance, it is possible HPA recognizes high Core 2 (GlcNAcβ1-6(Galβ1-3)GalNAcαSer/Thr) or Core 3 (GlcNAcβ1-6GalNAcαSer/Thr) expression on EL4 cells because the lectin has previously been reported as GlcNAc-specific [34,39], whereas Morniga G is not known for this. In addition, our comparative bioinformatic modeling of Morniga G and HPA suggest that in contrast to Morniga G, the HPA molecule should offer more possibilities for binding to branched *O*-glycans. Further experiments will need to be performed to demonstrate the involvement of Core 2 or Core 3 structures in HPA-mediated cell death.

Truncated *O*-glycans can strongly modulate T-cell function. Thus, beside the glycosylation on death receptors (such like DR5, DR4, or Fas) altered *O*-glycans carried by other glycoproteins can also be involved as possible targets for HPA. Indeed, numerous glycoproteins decorated by *O*-glycans are expressed on EL4 lymphoma [40,41], could be recognized by HPA and potentially involved in induction of cell death [42]. For instance, CD43 and CD45 glycoproteins are expressed by EL4 cells and carry many *O*-glycans [41]. In agreement with numerous observations, these cell-surface glycoproteins can be involved in cell death. CD43 and CD45 molecules are known to play a major role in T lymphocyte physiology [43]. The abundance and the structure of the *O*-glycans decorating CD45 and CD43 are modulated during activation and survival of T lymphocytes, and participate in the regulation of the life-span of T lymphocytes. For instance, activated T lymphocytes express CD45 carrying *O*-glycan Core 2 instead of *O*-glycan Core 1 that is present on naive T lymphocytes (i.e., T antigen) [44]. As a consequence of this change in *O*-glycosylation, the susceptibility to cell death inducers is modulated [43]. For instance, activated T lymphocytes become sensitive to galectin1-induced apoptosis [44] as compared to naive lymphocytes. Because certain surface markers of activated T-cells are also present on EL4 cells, this tumoral cell line could possibly be rich in Core 2 *O*-glycans and, from this point of view, more sensitive to HPA-induced cell death than naive T lymphocytes.

Although still poorly known, quantitative and qualitative modulations of *O*-glycans on the cell surface are normal phenomena in lymphocyte physiology [18,38,43]. The expression of truncated *O*-glycans on tumor cells could disturb the efficiency of anti-tumor immune responses. However, healthy naive T lymphocytes are Tn-negative, allowing the development of therapeutic targeting to eliminate Tn-positive tumor cells, or expressing other types of truncated *O*-glycans (such as Core 2 *O*-glycan). For instance, previously, in vitro experiments demonstrated that covalent conjugation of photosensitizers with MorG allows targeting towards aberrant glycans present on tumor cells, e.g., to purge leukemia cells from blood [45]. Several studies have indicated the possibility of using HPA and other H-type lectins for the specific delivery of drugs in tumor cells, e.g., for in vitro drug delivery to breast cancer cells [46]. However, to our knowledge, in vivo investigations using HPA (or MorG) have not been carried out to date in animal pre-clinical models.

## 4. Materials and Methods

### 4.1. Cell Cultures

Jurkat A3 human T-cell leukemia line (from ATCC, Manassas, VA, USA) was cultured in RPMI medium containing 10% FCS. EL4 mouse T-cell lymphoma line (ATCC, Manassas, VA, USA) was cultured in DMEM medium containing 10% FCS. Mouse splenocytes were extracted from C57BL/6 mice in DMEM medium, then splenocytes were incubated in DMEM medium on a Petri dish for two hours at 37 °C, to obtain non-adherent mouse splenocytes. C57BL/6 mice were a gift from Dr. A. Coste (UPS Toulouse University, Toulouse, France) and from the Anexplo platform. Mice were housed in temperature-controlled rooms in the specific pathogen-free animal facility (Anexplo platform, Toulouse, France), kept on a 12 h light/dark cycle, and had unrestricted access to food and water. All animal studies were conducted according to national and international policies.

### 4.2. Lectin-Mediated Cytotoxicity Assay and Cell Death Evaluation

HPA, Concanavalin A (Con A), and FITC-Con A were purchased from Sigma-Aldrich (Saint-Louis, MO, USA). FITC-HPA was purchased from Invitrogen (Carlsbad, CA, USA). Morniga-G (MorG) and Artocarpin (MorM) were purified from *Morus nigra* and *Artocarpus integrifolia*, respectively, as previously described [26,27]. Artocarpin and Morniga-G were labelled with FITC (Amersham Biosciences, Amersham, UK)

Jurkat A3 cells and EL4 cells were treated with Morniga-G, HPA, Artocarpin, and Concavalin A lectins for 48 h in RPMI or DMEM supplemented with SVF 10% at 37 °C/5% CO_2_ humidified atmosphere. Cell death was estimated by flow cytofluorometry analysis with Muse Annexin V and Dead Cell Assay kit (#MCH100105) using MUSE cell analyzer (Luminex Corporation, Austin, TX, USA). Cell death was also evaluated by fluorescent microscopy using DAPI death marker (#564907 BD Bioscience, Franklin Lakes, NJ, USA).

For mitochondrial membrane depolarization, cells were cultivated with lectins for 48 h and mitochondrial membrane potential (mitopotential) was analyzed using Muse MitoPotential Kit (#MCH100110) (Luminex Corporation, Austin, TX, USA) and Muse Cells Analyzer. Caspase activation were analyzed by Muse MultiCaspase Kit (#MCH100109).

### 4.3. Preparation of Healthy Lymphocytes and EL4 Mixtures

Non-adherent splenocytes, i.e., T, B, and NK lymphocytes, were prepared from C57BL6 mice as described above. EL4 cells were labeled with 1µg/mL CFSE (#65-0850-84 Invitrogen, Carlsbad, CA, USA) for 1 h and washed. Afterwards a mixture (1:1) was prepared with lymphocytes and CFSE-labeled EL4 cells in DMEM supplemented with SVF 10%. The mixture was cultured for 48 h in the presence of 20 μg/mL Morniga G or HPA. After washing, wells were incubated with PE-labelled anti-CD3 mouse monoclonal antibody (Becton Dickinson; Franklin Lakes, NJ, USA) and analyzed using cytofluorimetry.

### 4.4. Cell Surface-Binding Experiments

Cells (10^6^/mL) were incubated with FITC-Lectins (0.01 to 5 µg/mL), anti-Tn (anti-CD175), or anti-T (anti-CD176) mouse monoclonal antibodies (mAb) (1 µg/mL) (#MA1-90544 Invitrogen, Carlsbad, CA, USA) for 30 min at 4 °C in PBS. After incubation with mAb, cells were washed and stained with PE-conjugated secondary antibody (BD Bioscience) for 30 min at 4 °C in PBS. After washing, cells were analyzed using Fortessa FACS (BD Bioscience) flow cytometer. 7-AAD Viability Staining Solution (#00-6993-50 Invitrogen, Carlsbad, CA, USA) was added to exclude dead cells.

### 4.5. Neuraminidase Treatment of EL4 Cells

EL4 cells were treated with 20 mUI Neuraminidase (#E0540S New England Biolabs, Ipswich, MA, USA) for 30 min at 37 °C in PBS, then washed before using.

### 4.6. ROS Detection

EL4 cells were exposed to HPA or Concanavalin A lectins in DMEM supplemented with SVF 10% at 37 °C/5% CO_2_ humidified atmosphere. ROS production was analyzed by Muse Oxidative Stress Kit (#MCH100111) or Muse Nitric Oxide Kit (#MCH100112) using Muse Cells Analyzer. ROS were also analyzed by dihydroethidium (DHE) fluorescent probe using Fortessa FACS (BD Bioscience, Franklin Lakes, NJ, USA).

### 4.7. Statistical Analyses

Results are expressed as the means ± SD of data obtained from at least three independent experiments. Statistical significance was determined by means of Student’s *t*-test. *p* < 0.05 was considered significant.

### 4.8. In Silico Molecular Modeling and Docking Experiments

Homology modelling of Morniga-G, was performed with YASARA [47], using the X-ray coordinates of frutalin from *Artocarpus incisa* (PDB code 4WOG), jacalin from *Artocarpus heterophyllus* (PDB code 3P8S), the *Maclura pomifera* agglutinin MPA (PDB code 3LLZ) [48], and agglutinin a-chain from *Artocarpus integer* (PDB codes 1UGX and 4AK4) [49,50], as templates. Five different models were built for Morniga-G and finally, a single hybrid model was built up from the previous models. PROCHECK [51], ANOLEA [52], and the calculated QMEAN scores [53,54], were used to assess the geometric and thermodynamic qualities of the Morniga-G three-dimensional model. A single amino acid residue D163 on a total of 166 residues, occurred in the non-allowed regions in the Ramachandran plot. Using ANOLEA to evaluate the Morniga-G model, only 17 residues (out of 166) exhibited an energy over the threshold value. The calculated QMEAN score gave an acceptable value of 0.96. The three-dimensional structure of *Helix pomatia* agglutinin (HPA) was taken from the PDB (PDB code 2CE6 for the unliganded lectin, PDB codes 2CCV for HPA in complex with GalNAc and 2CGZ for HPA in complex with Tn antigen) [36,55].

Docking of GalNAc, GlcNAc, and Tn antigen was performed with YASARA. Some docking experiments were performed at the SwissDock web server (http://www.swissdock.ch, accessed on 21 May 2021) [56,57], as a control for our docking experiments. Molecular cartoons were drawn with Chimera [58] and Chimera-X [59].

## 5. Conclusions

In conclusion, comparative experiments with Morniga G and HPA, two exogenous lectins known to be T/Tn-specific, demonstrated similar effects on Tn-positive leukemic lymphocytes. However, only HPA killed T/Tn-negative T lymphoma cells efficiently without affecting healthy lymphocytes. This observation suggests that HPA is not only a good tool as cancer marker, but could also be used for therapeutic applications.

## Data Availability

Data is contained within the article.
